# Role and molecular mechanism of ghrelin in degenerative musculoskeletal disorders

**DOI:** 10.1111/jcmm.17944

**Published:** 2023-09-03

**Authors:** Jianfeng Sun, Yibo Tan, Jingyue Su, Herasimenka Mikhail, Volotovski Pavel, Zhenhan Deng, Yusheng Li

**Affiliations:** ^1^ Deparment of Orthopedics Xiangya Hospital, Central South University Changsha Hunan China; ^2^ Xiangya School of Medicine, Central South University Changsha Hunan China; ^3^ Department of Sports Medicine The First Affiliated Hospital of Shenzhen University, Shenzhen Second People's Hospital Shenzhen Guangdong China; ^4^ Republican Scientific and Practical Center of Traumatology and Orthopedics Minsk Belarus; ^5^ National Clinical Research Center for Geriatric Disorders Xiangya Hospital, Central South University Changsha Hunan China

**Keywords:** ageing, ghrelin, intervertebral disc degeneration, osteoarthritis, osteoporosis, sarcopenia

## Abstract

Ghrelin is a brain‐gut peptide, and the first 28‐peptide that was found in the gastric mucosa. It has a growth hormone (GH)‐releasing hormone‐like effect and can potently promote the release of GH from pituitary GH cells; however, it is unable to stimulate GH synthesis. Therefore, ghrelin is believed to play a role in promoting bone growth and development. The correlation between ghrelin and some degenerative diseases of the musculoskeletal system has been reported recently, and ghrelin may be one of the factors influencing degenerative pathologies, such as osteoporosis, osteoarthritis, sarcopenia and intervertebral disc degeneration. With population ageing, the risk of health problems caused by degenerative diseases of the musculoskeletal system gradually increases. In this article, the roles of ghrelin in musculoskeletal disorders are summarized to reveal the potential effects of ghrelin as a key target in the treatment of related bone and muscle diseases and the need for further research.

## 1 INTRODUCTION

Ghrelin, a 28‐peptide, is produced mainly by P or D1 cells at the bottom of the human stomach and by epsilon cells of the pancreas. It was first discovered by Kojima in 1999 as an endogenous ligand for the growth hormone secretagogue receptor (GHSR)1a, which is capable of stimulating the release of growth hormone (GH) from the anterior pituitary gland.^1^ Recent studies^2^, ^3^, ^4^ have proven that although ghrelin is a peptide present in the digestive system, it plays a role in promoting the growth and development of bones and exerts chondroprotective and anti‐inflammatory effects on joints. Ghrelin also regulates the central sympathetic nerve activity.^5^

Degenerative musculoskeletal disorders, including osteoarthritis (OA), osteoporosis (OP), sarcopenia and intervertebral degenerative disc disease (IVDD), have been associated with ageing and inflammation.^6^, ^7^ Since most degenerative disorders occur in elderly individuals, the challenge of global ageing may lead to an increase in both the prevalence and social costs related to these disorders.^8^ OP has a high incidence worldwide,^9^, ^10^ and the cost of OP‐related fractures to the global economy is expected to rise to $131.5 billion by 2050.^11^ The two different types of OP are primary and secondary OP, with the former being more prevalent in clinical practice and mainly affecting elderly people and postmenopausal women.^12^, ^13^ OA is the most common form of arthritis that affects 1 in 3 older adults and has a greater impact on women than on men.^14^, ^15^ In the United States, Canada, the United Kingdom, France and Australia, it is estimated that approximately 1.0%–2.5% of the gross domestic product was contributed by economic activities related to the treatment of musculoskeletal conditions, including OA.^16^ Approximately 80% of the costs incurred by musculoskeletal diseases are associated with symptomatic OA. Sarcopenia is a disease closely related to ageing,^17^ whose prevalence can be as high as 50% in individuals aged >80 years.^18^ Sarcopenia can result in a substantial loss of muscle quality and function, and patients, especially elderly individuals, may suffer from a higher risk of fall with serious injuries, troubles in standing and walking, and danger of losing self‐care capabilities, placing a significant burden on their family and society.^19^ IVDD is a chronic condition that can be induced by several factors and represents an important cause of morbidity and mortality in everyday clinical practice.^20^ Moreover, it is well known that IVDD is correlated with low back pain (LBP),^21^ while LBP is estimated to have direct costs of up to $90 billion annually in the United States alone.^22^ The overall socioeconomic effects of IVDD bring over a significant problem for the society, especially when considering the indirect costs associated with disability.^23^

Thus, degenerative musculoskeletal disorders pose significant challenges to human health. Over the past decade, several studies have highlighted the importance of ghrelin in the treatment of various diseases. As ghrelin has been scarcely studied in degenerative musculoskeletal disorders, this article aimed to comprehensively review the roles and molecular mechanisms of ghrelin in various degenerative musculoskeletal disorders, such as OA, OP, sarcopenia and IVDD.

## 2 GHRELIN

Ghrelin was first discovered and named by Kojima et al.^1^ The activation of ghrelin requires a series of unique modifications, including acylation of the third serine residue catalysed by ghrelin acyltransferase. Ghrelin is a peptide that circulates in both acylated and unacylated forms. Acylated ghrelin is an active form that binds to the typical ghrelin receptor, GHS‐R1a, whereas unacylated ghrelin is not capable of binding to this receptor. GHS‐R1a and b are two ghrelin receptors, with the former recognized as a functional receptor. GHS‐R1a consists of 366 amino acids (AAs) with seven transmembrane domains (TMDs 1–7),^24^ while GHS‐R1b consists of 289 AAs with the TMDs 1–5 of GHS‐R1a and a part of the connected intron. GHS‐R1a is the only inducer of intracellular Ca2C signalling that triggers the activation of a G‐protein subtype, Gaq/11, in response to an agonist.^1^, ^24^, ^25^ In contrast, because of the absence of TMDs 6 and 7, GHS‐R1b does not induce Ca2C signalling. GHS‐R is associated with the G(q) and G(s) signalling pathways, and the intracellular calcium concentration is elevated as a result of the binding between ghrelin or synthetic peptidyl and nonpeptidyl ghrelin mimetic agents.

Ghrelin modulates systemic metabolism via the activation of orexigenic neural circuits.^26^, ^27^ Several central and peripheral actions of ghrelin have been identified, including the stimulation of gut motility and gastric acid secretion,^28^, ^29^ modulation of sleep,^30^ regulation of glucose metabolism,^31^, ^32^ suppression of brown fat thermogenesis,^33^ prevention of muscle atrophy,^34^, ^35^ promotion of bone growth and development^2^ and exertion of chondroprotective and anti‐inflammatory effects on joints.^3^, ^4^

## 3 GHRELIN AND AGEING

Ghrelin secretion is age‐dependent, and its concentration may be reduced by changes in the body composition associated with ageing, such as the loss of body fat and muscle. It has been reported that not only do fasting ghrelin levels depend on age,^36^ but the decrease in plasma ghrelin concentration also correlates with age. The blood concentration of ghrelin in healthy elderly individuals was 20%–35% lower than that in young individuals.^37^, ^38^ Increased ghrelin adaptations were observed in young malnourished patients but not in elderly ones, suggesting that ghrelin may play a role in the increased incidence of malnutrition in elderly individuals.^39^ In addition, acylated ghrelin circulates in lower concentrations and there is a significantly weaker association between the circulating acylated ghrelin levels and GH secretion in elderly people.^40^ The adrenergic hormones (norepinephrine and epinephrine) can specifically stimulate ghrelin secretion.^41^ The increased secretion of ghrelin in vivo is due to sympathetic stimulation^42^, ^43^ or local infusion of adrenergic hormones into the gastric lining.^44^ The intravenous infusion of epinephrine does not increase plasma ghrelin,^42^ indicating that adrenergic agents directly acted on β1 receptors in ghrelin‐secreting cells. Several studies have found that the β‐adrenergic reactivity shows a decreasing trend with age,^45^ which may contribute to the reduced acylated ghrelin levels in elderly individuals.

A decrease in the plasma ghrelin concentration may be associated with the altered GH function and/or anorexia nervosa in elderly individuals.^38^ The fasting acylated ghrelin concentration is lower in elderly individuals. After a meal, acylated ghrelin reaches its lowest concentration in young individuals and increases to the fasting concentration 2–4 h later, showing pulsatile dynamics. In elderly individuals, the postprandial acylated ghrelin concentration remains low after a meal.^46^ Age‐related changes in body composition, such as a decrease in muscle and an increase in fat mass, may lead to reductions in fasting and postprandial ghrelin concentrations because the body fat content is negatively correlated with ghrelin concentration and increases with age.^47^

Ghrelin was found in the stomach, other parts of the gut and, indeed, in all the tissues studied (adrenal gland, atrium, breast, buccal mucosa, oesophagus, fallopian tube, fat tissue, gall bladder, human lymphocytes, ileum, kidney, left colon, liver, lung, lymph node, muscle, myocardium, ovary, pancreas, pituitary, placenta, prostate, right colon, skin, spleen, testis, thyroid and vein).^48^ Unlike ghrelin, which is widely expressed, the expression of GHS‐R is considerably restricted in terms of both location and content.^33^, ^48^, ^49^, ^50^ A recent study^51^ demonstrated that the GHS‐R mRNA expression was elevated in ageing muscles and was positively linked with losses of muscle mass and metabolic dysfunction, indicating that ghrelin signalling may be implicated in age‐related functional decline.

The effect of ghrelin on the bone structure gradually shifts from a systemic/central stimulatory effect to a local inhibitory effect with an increasing age.^52^ The increased osteoclastogenesis and reduced bone mass in aged GHSR–/− mice suggested that the systemic stimulation pathway was weakened with ageing, thereby allowing the local inhibitory effect of ghrelin to predominate. This age‐dependent relationship between ghrelin and bones is reversed during ageing. Ghrelin was not associated with bone resorption markers in a cohort of adolescent boys^53^ or with bone mass in young men,^54^ whereas a negative association with bone resorption and a positive association with bone resorption mass were observed in a large sample‐based study of elderly men.^55^

Ghrelin also stimulates GH release. It exerts peripheral effects on the pituitary gland and hypothalamus by regulating the vagus nerve. Ghrelin acts on somatotrophs of the anterior pituitary gland, GH‐releasing hormone (GHRH)‐secreting neurons and GH‐inhibiting hormone (GHIH)‐secreting neurons in the hypothalamus (against the action of somatostatin) through G protein‐coupled receptor GHS‐R1a.^56^, ^57^ Additionally, the GH response to ghrelin shows a significant age‐related declining trend.^58^ The age‐related decrease in GH secretion reflects changes in the neurologically controlled GH function, which include a concomitant decrease in GHRH and an increase in GHIH activity.^59^ This explains that the human response to ghrelin and GHS gradually reduces with ageing. In brief, ageing is negatively correlated with plasma ghrelin concentrations; additionally, decreased fasting and postprandial ghrelin concentrations in elderly individuals may be attributed to an increase in body fat, decrease in GHRH and increase in GHIH activity.

## 4 GHRELIN AND DEGENERATIVE DISEASES

**FIGURE 1:**
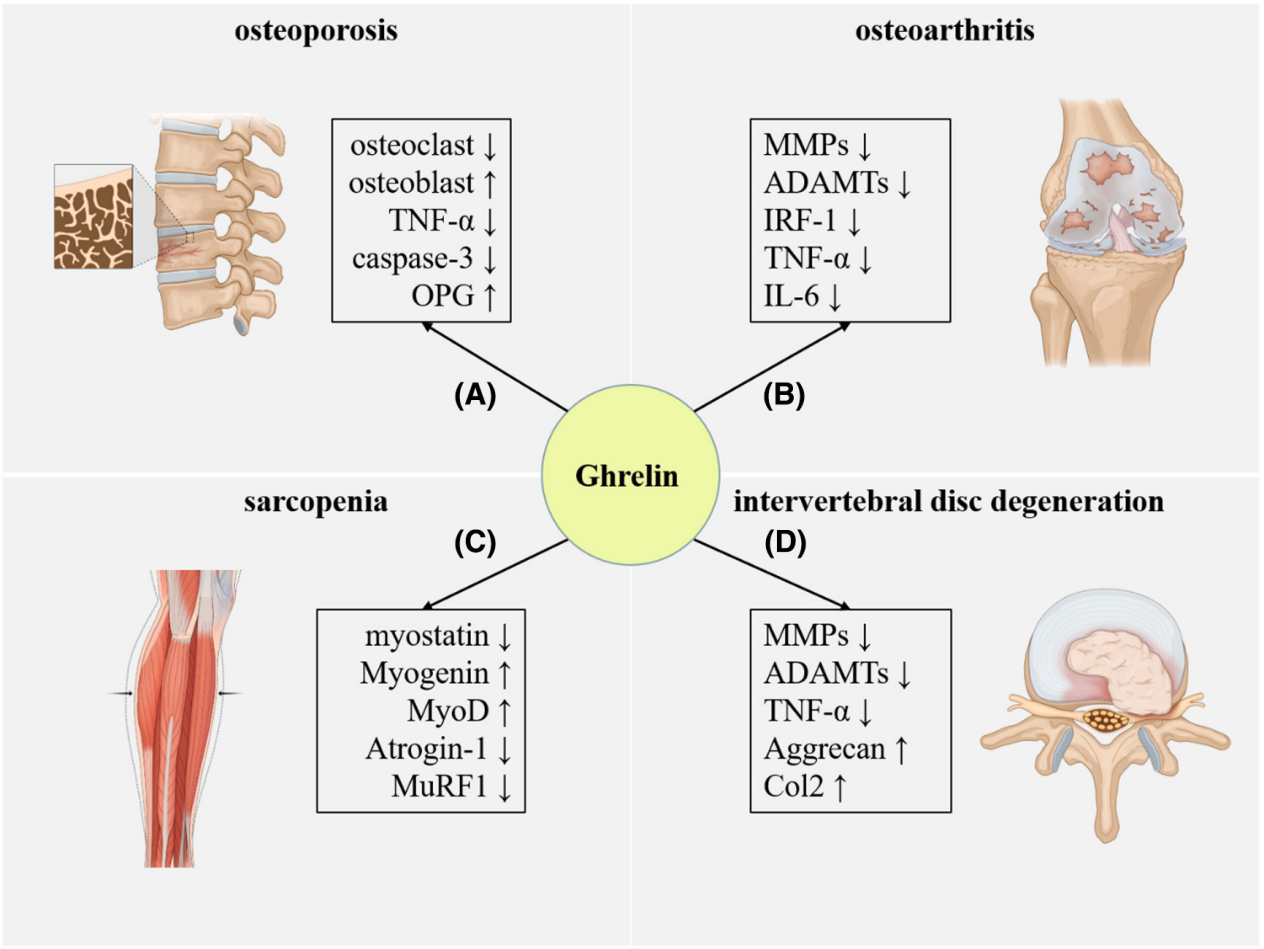
Ghrelin is correlated with the progression of multiple degenerative musculoskeletal disorders including OP, OA sarcopenia and IVDD. (A) Signal pathway: MAPK/PI3K, GHSR/ERK and GHSR/PI3K/AKT. (B) Signal pathway: JAK2/STAT3/IRF‐1, NF‐kB and AKT. (C) Signal pathway: p38/C/EBPβ, PI3Kβ, mTORC2 and p38. (D) Signal pathway: NF‐κB and AKT. AKT, protein kinase B; ERK, extracellular regulated protein kinase; GHSR, growth hormone secretagogue receptor; IVDD, intervertebral disc degeneration; MAPK, mitogen‐activated protein kinase; mTORC2, mammalian target of rapamycin complex 2; OA, osteoarthritis; OP, osteoporosis; PI3K, phosphoinositide 3‐kinase; PI3Kβ, phosphoinositide 3‐Kinase β.

Ghrelin was first found in the gastrointestinal tract, where it is highly expressed before eating; however, its plasma level drops dramatically postprandially. However, recent studies^2^, ^3^, ^4^ have found that ghrelin not only acts as a gastrointestinal peptide in the digestive system, but also plays an important role in the musculoskeletal system. The associations between ghrelin levels and degenerative musculoskeletal disorders are shown in Figure 1 and Table 1.

**TABLE 1:** Roles of ghrelin in degenerative musculoskeletal disorders.

Disease	Signal pathway	Roles in disease	References
OP	MAPK/PI3K	Promoting osteoblast proliferation and differentiation and inhibiting apoptosis	[57], [58]
	GHSR/ERK and GHSR/PI3K/AKT	Inhibiting apoptosis in MC3T3‐E1 cells	[59]
	GH‐IGF‐I axis	Increasing BMD	[60]
OA	JAK2/STAT3/IRF‐1	Suppressing the expression of MMPs and ADAMTs and inhibiting degradation of human chondrocyte aggregates and Col II	[61], [62]
	NF‐κB	Antagonizing exaggerated catabolism in degenerative chondrocytes	[63]
	AKT	Suppressing the disorganized anabolism	[63]
Sarcopenia	p38/C/EBPβ	Inhibiting myostatin activation	[64]
	PI3Kβ, mTORC2, and p38	Inhibiting dexamethasone‐induced skeletal muscle atrophy and atrogene expression	[34]
IVDD	NF‐κB	Inhibiting the role of IL‐1β in NP cell proliferation and apoptosis	[65]
	AKT	Promoting anabolism in NP cells	[65]

Abbreviations: ADAMTs, a disintegrin and metalloproteinase with thrombospondin motifs; AKT, protein kinase B; BMD, bone mineral density; ERK, extracellular regulated protein kinase; GH, growth hormone; GHSR, growth hormone secretagogue receptor; IGF, insulin‐like growth factors; IL‐1β, interleukin‐1β; IRF‐1, interferon regulatory factor‐1; IVDD, intervertebral disc degeneration; MAPK, mitogen‐activated protein kinase; MMPs, matrix metalloproteinases; mTORC2, mammalian target of rapamycin complex 2; NP, nucleus pulposus; OA, osteoarthritis; OP, osteoporosis; PI3K, phosphoinositide 3‐kinase; PI3Kβ, phosphoinositide 3‐Kinase β.

### 4.1 Ghrelin and OP

Ghrelin has been shown to promote the growth and differentiation of osteoblasts and inhibit osteoclast production, thereby exerting effects on bone metabolism by promoting bone formation and suppressing bone resorption.^66^, ^67^ Ghrelin can directly stimulate the proliferation and differentiation of mouse osteoblasts, MC3T3‐E1 and exert antiapoptotic effects on MC3T3‐E1 cells under both pharmacological and physiological conditions. Both acylated and unacylated ghrelin promote the growth of human osteoblasts via the mitogen‐activated protein kinases/phosphoinositide 3‐kinase (PI3K) pathway in the absence of GHS‐R1a.^68^ Ghrelin may play a role in regulating the activity of insulin‐like growth factor 1 (IGF‐1) in osteoblasts, thereby significantly reducing the apoptosis of osteoblasts induced by tumour necrosis factor‐alpha (TNF‐α) and inhibiting the activation of caspase‐3, which makes it the main contributor to the mechanism of apoptosis in several cell types.^67^, ^68^ It was also found that blocking extracellular signal‐regulated kinase (ERK) and AKT (also called protein kinase B [PKB]) and inhibiting GHSR could both block the protective effect of ghrelin against desmosome‐induced apoptosis in MC3T3‐E1 cells. This finding may suggest that ghrelin inhibits apoptosis in MC3T3‐E1 cells by activating the GHSR/ERK and GHSR/PI3K/AKT signalling pathways.^60^ It further hints that ghrelin may affect bone metabolism by controlling osteoblast apoptosis. Moreover, ghrelin was revealed to have mitogenic activity in osteoblasts, with a stronger effect on human cells and a weaker one on rat osteoblasts.^69^ Ghrelin and GHS‐R1a have been shown to promote osteoblast proliferation and differentiation in vitro and increase the bone mineral density in vivo.^66^ Furthermore, ghrelin can significantly increase the number of osteoblasts and the synthesis of DNA in a dose‐dependent manner. It also increases the expression of osteoblast differentiation markers, alkaline phosphatase activity and calcium accumulation in the matrix.^66^ Collectively, ghrelin plays an important role in promoting the proliferation and differentiation of osteoblasts.

Conversely, ghrelin has an inhibitory effect on osteoclast formation.^70^ It can promote osteoprotegerin (OPG) expression while inhibiting the receptor activator of the nuclear factor kappa‐B ligand/OPG ratio, resulting in decreased osteoblast‐related osteoclast production.^71^ Ghrelin‐mediated inhibition of osteoclastogenesis and reduction of bone loss is age‐dependent. Both osteoblasts and ‐clasts express mRNAs encoding ghrelin and its receptor, and their expression levels decline with age. The role of ghrelin in promoting the proliferation and differentiation of osteoblasts^67^, ^68^ and directly inhibiting osteoclasts^52^ may be one of the reasons for the increased bone resorption with ageing, leading to OP. When the inhibitory effect on osteoclasts is weakened and the proliferation and differentiation of osteoblasts is reduced, bone resorption is stronger than bone formation, resulting in bone loss and OP.

Bone mineral density (BMD) can be used as a monitoring indicator for OP, while ghrelin, a natural ligand for GHSR and GH, can increase BMD.^2^ Therefore, it is speculated that ghrelin affects BMD through GH‐related pathways. Furthermore, ghrelin injections in rats were observed to significantly increase the total BMD of the femur.^72^ The same experiment was performed in GH‐deficient mice to exclude ghrelin from activating the GH‐IGF‐I axis, and it was found that ghrelin also increased BMD in mice. Additionally, it was proposed that acylated ghrelin might be a significant determinant of whole‐body BMD in healthy normal‐weight children and adolescents.^66^ Therefore, while ghrelin is highly likely to directly stimulate an increase in BMD, it can also increase it by stimulating GH release.

The effects of body weight on bone formation, bone resorption and serum ghrelin have been reported by several investigators.^73^ It was shown that bone formation is inhibited and bone resorption promoted in females after a 10% weight loss, and this bone conversion is influenced by the ghrelin level in the circulating blood. In addition, changes in the bone formation marker procollagen type I N‐propeptide have been reported to be positively correlated with changes in the lean body mass and negatively correlated with changes in serum ghrelin.^73^ Due to ageing and weight loss in elderly individuals, bone formation is inhibited and bone resorption is promoted, which jointly results in OP, while a decrease in the level of ghrelin or active ghrelin in the body can affect the level of GH.

Moreover, the serum ghrelin levels were positively correlated with trabecular bone density. In women, it was found that trabecular bone density increased by an average of 7.1 mg/cm^3^ for every 0.05 ng/mL increase in the ghrelin serum concentration, and the ghrelin level was highly correlated with trabecular bone density in elderly individuals.^61^ Ghrelin cells in the oxyntic mucosa are closed types of cells that are not continuous with the lumen of the gastrointestinal tract but are located adjacent to the capillary network of the lamina propria. They respond to physical stimuli from the lumen or chemical stimuli from the basolateral site. Therefore, they can function as endocrine cells to deliver ghrelin to the peripheral tissues expressing GHS‐R.^62^ Acylated ghrelin can affect osteoblasts and osteoclasts, while unacylated ghrelin has biological activities that specifically regulate the development and growth of osteoblasts.^70^ Acylated and unacylated ghrelin are indistinguishable in controlling the cortical bone mass, mineral deposition and bone formation in vivo, and in the formation of osteoblast colonies in vitro. The absence of GHSR‐mediated acylated ghrelin signalling can be compensated by unacylated ghrelin signalling, and intact ghrelin signalling protects early trabecular bone formation. Unlike the effect on osteoblasts, enhanced osteoclast formation was observed in GHRl–(no acylated and unacylated ghrelin response) and GHSR–(no acylated ghrelin response) deficient mice, suggesting that the formation of osteoclasts is primarily controlled by acylated ghrelin.

### 4.2 Ghrelin and OA

OA occurs because of cartilage damage and degeneration caused by inflammatory factors. Recent studies have shown that ghrelin has cartilage‐protective and anti‐inflammatory effects,^63^ suggesting that decreased ghrelin levels may be associated with OA development.

A strong association has been reported between a reduced expression of synovial fluid ghrelin and the progression of joint inflammation and cartilage damage.^74^ Under several pathological circumstances, ghrelin has demonstrated its anti‐inflammatory and antioxidant effects.^4^ Matrix metalloproteinases (MMP), a disintegrin and metalloproteinase with thrombospondin motifs (ADAMTS), interleukin‐8 (IL‐8) and complement factors are all induced by IL‐1, which regulates the degradation of the cartilage matrices.^75^ Ghrelin can ameliorate the IL‐1β‐induced degradation of type II collagen (Col II) and aggrecan,^76^ among which aggrecan degradation is another important pathological process in OA and is mainly regulated by ADAMTS‐4 and 5. Ghrelin inhibits IL‐1β‐induced expressions of ADAMTS‐4 and 5 at both the gene and protein levels. Moreover, MMP‐3 and MMP‐13 are associated with Col II degradation. Ghrelin inhibits the expression of MMP‐3, MMP‐13, ADAMTS‐4 and ADAMTS‐5 induced by IL‐1β in a concentration‐dependent manner, thereby reducing the degradation of Col II and aggrecan.

The expression of interferon regulatory factor 1 (IRF‐1) was reported to increase in the chondrocytes of patients with OA, and immunofluorescence staining indicates that IL‐1β promotes the expression of IRF‐1.^77^ Polymerase chain reaction (PCR) results show that ghrelin attenuates the IL‐1β‐induced increase in the expression of IRF‐1 mRNA. Ghrelin can inhibit IRF‐1 expression by inactivating the Janus kinase 2 (JAK2)/signal transducer and activator of the transcription 3 (STAT3) pathway.^76^ It was found that ghrelin inhibits the expressions of MMP‐3, MMP‐13, ADAMTS‐4 and ADAMTS‐5 and suppresses the activation of the JAK2/STAT3 pathway, but not the p38 pathway.^77^ Overall, ghrelin weakens the ability of IL‐1β‐induced degradation of human chondrocyte aggregates and Col II and suppresses the expressions of MMPs and ADAMTs by inhibiting the JAK2/STAT3/IRF‐1 pathway.

Ghrelin can reduce the production of several inflammatory cytokines, inhibit chondrocyte apoptosis, downregulate the levels of MMP‐13 and ADAMTS‐5 and maintain critical matrix components, such as aggrecan and Col II to inhibit cartilage degeneration by safeguarding the dynamic balance of chondrocytes.^78^ Among the various degenerative and inflammatory cytokines involved in OA, IL‐1β and TNF‐α are the key proinflammatory cytokines that induce other inflammation‐related molecules.^79^, ^80^ In the development of OA, activated AKT signalling would increase chondrocyte anabolism, while inhibited AKT signalling in chondrocytes would reduce chondrocyte anabolism.^81^ An important modulator of age‐dependent cartilage degradation is the nuclear factor kappa‐light‐chain enhancer of the signalling pathway of activated B cells (NF‐κB).^82^ Studies have shown that the activation of NF‐kB signalling can accelerate the progression of age‐related cartilage degenerative diseases, while inhibition of this signalling can attenuate these diseases.^83^, ^84^ Ghrelin was found to antagonize the inhibition of the chondrocyte AKT signalling pathway and activation of NF‐κB signalling during OA development, alleviate disordered anabolism, inhibit inflammatory cytokines or OAs‐induced hyperactivation of NF‐κB signalling in chondrocytes and downregulate IL‐1β‐mediated catabolism. Moreover, the expression levels of ghrelin in degenerative cartilage, inflammatory cytokine‐stimulated chondrocytes and cartilage in a surgically induced arthritis model were significantly reduced, suggesting that ghrelin may be involved in the development of OA.^78^ In two types of surgically induced OA models, both systemic and topical applications of ghrelin significantly reduced the disease severity, suggesting that it may play a protective role in articular cartilage degeneration.^78^

Alternatively, the ghrelin level in synovial fluid is associated with disease severity and ghrelin is negatively correlated with the TNF‐α and IL‐6 levels.^85^ Ghrelin can potently inhibit the expressions of TNF‐α, IL‐1β and IL‐6 by lymphocytes and monocytes.^86^ Increased endogenous ghrelin was reported to downregulate the pain threshold and exert antinociceptive effects to alleviate inflammatory pain in rats by interacting with the central opioid system,^64^, ^87^ suggesting that ghrelin may suppress inflammation‐induced pain.

Ghrelin plays an important role in maintaining the normal function of the musculoskeletal system. The reduction in ghrelin levels in the body weakens its protective effects on the cartilage and its anti‐inflammatory effects, which can lead to symptoms of OA. These findings support the feasibility of ghrelin as a future treatment for OA.

### 4.3 Ghrelin and sarcopenia

Sarcopenia is a disease that seriously endangers the physical health and behavioural movement of elderly individuals with the loss of skeletal muscle mass and strength, which has brought about heavy socioeconomic pressure as the population ages. Muscle mass declines with age,^88^ and is significantly reduced in the elderly.^89^ With ageing, a certain degree of skeletal muscle degeneration and sarcopenia occurs, and ghrelin, as a gastrointestinal hormone, plays an important role in maintaining the muscle function.

Ghrelin has been proven to be capable of preventing muscle catabolism, promoting intestinal motility and regulating metabolism.^90^, ^91^ The protective effect of ghrelin can be attributed to two aspects: It stimulates feeding to increase the intake of protein and other nutrients,^92^ and it has a direct protective effect on muscles by preventing muscle breakdown through the regulation of metabolic factors.

It was found that both acylated and unacylated ghrelin stimulated C2C12 skeletal myoblasts to differentiate and fuse to form multinucleated myotubes in vitro by activating the p38 pathway.^35^ Myostatin is a key regulator of skeletal muscle mass and is expressed in skeletal muscle to suppress muscle growth.^93^ Ghrelin and its analogues Bim‐28,125 and Bim‐28,131 were found to significantly reduce the expression of myostatin at the protein level,^92^ suggesting that ghrelin may exert a protective effect on muscle growth against inhibition by myostatin. p38 mediates effects through C/EBP‐β, and C/EBP‐β activation can induce the expression of myostatin.^94^ In C2C12 cells, it was reported that cisplatin elevated the nuclear C/EBP‐β and myostatin, confirming that the p38/C/EBP‐β/myostatin pathway was activated in this situation.^91^ Ghrelin may inhibit myostatin activation by inactivating p38 and C/EBPβ and act directly on myoblasts to prevent cisplatin‐induced myasthenia in the absence of GHSR1a,^91^ suggesting the possibility that ghrelin may play a role in preventing myasthenia gravis or sarcopenia.

In cultures of GHSR‐1a‐deficient C2C12 myotubes, acylated and unacylated ghrelin can trigger antiatrophic signalling so as to protect the myotubes against dexamethasone‐induced atrophy and atrogene overexpression.^34^ Moreover, TNF/IFN‐induced cachexia in C2C12 myotubes was reported to be reduced by unacylated ghrelin in a PI3K/ mammalian target of rapamycin (mTOR)‐dependent manner.^95^ It has been shown that the mTOR Complex 2 (mTORC2) plays a crucial role in regulating the antiatrophic effects of acylated ghrelin and unacylated ghrelin since their antiatrophic activity is sensitive to the downregulation of rictor, a specific mTORC2 component. Acylated and unacylated ghrelin inhibit the dexamethasone‐induced skeletal muscle atrophy and atrophy gene expression through PI3Kβ, mTORC2 and p38‐mediated myotubular pathways. They are unaffected by GHSR‐1a or stimulation of the GH/IGF‐1 axis.^34^ Acylated and unacylated ghrelin were also found to activate an antiatrophic signalling pathway by directly acting on skeletal muscle, protecting it against experimentally induced atrophy. In addition, ghrelin can act on a common, unknown receptor to prevent myasthenia gravis in a GH‐independent manner and prevent the development of sarcopenia.^34^ These findings suggest that ghrelin may be an important contributor to prevent muscle atrophy and promote muscle growth.

The myogenic regulatory factors, myogenic differentiation factor (MyoD) and myogenin, which are controlled by AKT phosphorylation, p38, myostatin and TNF‐α, have been demonstrated to play a significant role in tumour‐induced cachexia by controlling muscle regeneration.^96^, ^97^ Ghrelin was found to be capable of blocking the changes caused by cisplatin, leading to elevated expressions of atrogin‐1, muscle ring finger (MuRF)1, p38 and myostatin and reduced expressions of AKT, MyoD and myogenin.^91^ Muscle atrophy occurs through the lysosomal and protease systems by degrading large molecules of protein within the myocyte when the body is in a state of nutritional deficiency. The ubiquitin–protease system is the major protein degradation system, and the genes encoding atrogin‐1 and MuRF1 are critical genes that control protein degradation.^98^ The activation of p38/C/EBP‐β, myostatin and inflammatory cytokines, as well as the decrease in AKT and myogenin/myoD, would eventually lead to increased proteolysis, decreased muscle mass and reduced strength. Overall, ghrelin inhibits muscle atrophy by activating AKT, myogenin and myoD and downregulating inflammation and p38/C/EBP‐β/myostatin. These alterations are speculated to target muscle cells directly, at least in part.

### 4.4 Ghrelin and IVDD

IVDD is a chronic and complex degenerative lesion of the locomotor system characterized by metabolic and structural changes, which can result in degeneration, loss of mechanical stability, and shock‐absorbing function of the intervertebral disc (IVD).^99^ Inflammation is an extremely vital factor in IVDD, which is a process that can be exacerbated by the production of inflammatory mediators. Meanwhile, the production of mediators, including IL‐1β, TNF‐α and IL‐6 has been associated with matrix degradation, ageing and death of disc cells, as well as the recruitment of immune cells.^100^, ^101^ Ghrelin has anti‐inflammatory effects, which make it possible to contribute to the protection of IVDs against degeneration.

An important characteristic change in the degenerative process of IVD is the reduced anabolism of nucleus pulposus (NP) cells.^102^ In healthy, hydrated and mechanically functional IVDs, cells of the NP region are principally responsible for producing a functional extracellular matrix (ECM) and secreting chemokines and growth factors that control matrix formation.^103^, ^104^ Col II and aggregates are the key structural components of the cartilage and NP cells,^105^ and a decrease in these matrix molecules indicates excessive degeneration of the IVD.^106^ IL‐1β is known to be a key inflammatory factor that stimulates the expressions of several degenerative MMPs and polymerases, such as ADAMTS‐5, MMP‐13 and disintegrin.^107^, ^108^, ^109^ It has been widely recognized that IL‐1β is a major factor in the deterioration of IVD tissue.^110^ The ghrelin level in NP cells was reported to decrease after IL‐1β stimulation, suggesting a potential link between ghrelin and homeostasis in NP cells.^111^ In IVDD, it is common to observe elevated expressions of degeneration‐related molecules, including ADAMTS‐5, MMP13, iNOS and TNF‐α in NP cells.^112^, ^113^, ^114^ In a rabbit IVDD model, ghrelin administration was found to downregulate the elevated levels of degeneration‐related biomarkers, such as ADAMTS‐4, ADAMTS‐5 and MMP‐3 in NP tissues.^65^

The NF‐B pathway has been identified as a significant mediator of age‐dependent disc degeneration and is crucial for mediating IL‐1β activity.^115^ It was reported that ghrelin significantly slows down the nuclear transaction of p65, a known indicator for activation of the NF‐B signalling pathway.^111^, ^116^ Ghrelin can also suppress the excessive activation of the NF‐B signalling pathway in the presence of IL‐1, which may slow down the process of NP degeneration.^111^ Additionally, ghrelin is known to activate the AKT signalling pathway,^117^ which is directly related to the anabolic capacity of NP cells.^118^ Aggrecan and Col II are the key matrix components of NP tissues, and ghrelin can significantly upregulate the mRNA levels of aggrecan and Col II in a dose‐dependent manner.^111^

In general, ghrelin inhibits metabolic disorders and the apoptosis of NP cells by inhibiting the IL‐1β‐induced NK‐κB signalling pathway and stimulates the anabolic metabolism of NP cells by activating the AKT signalling pathway, thus providing a protective effect on IVD.

## 5 CONCLUSION

Ghrelin is mainly produced in the gastrointestinal tract and is speculated to play an important role in the musculoskeletal system based on the accumulated evidence. As age advances, ghrelin production and the proportion of active ghrelin show declining trends. Ghrelin has anti‐inflammatory and regulatory effects on bone metabolism and protects against degeneration in the musculoskeletal system. More specifically, ghrelin inhibits the production of osteoclasts and promotes the growth and differentiation of osteoblasts by acting on the GHSR/ERK and GHSR/PI3K/AKT signalling pathways. The chondroprotective and anti‐inflammatory effects of ghrelin are enabled by inhibiting the JAK2/STAT3/IRF‐1 and chondrocyte AKT signalling pathways and activating the NF‐κB signalling pathway, which makes ghrelin an important component in the protection of bones and joints. In terms of muscle protection, ghrelin promotes the intake of nutrients and acts directly on muscles to promote the proliferation of C2C12 myoblasts by activating AKT, myogenin and myoD while downregulating inflammation and p38/C/EBP‐β/myostatin. In the protection of IVD, ghrelin inhibits the IL‐1β‐induced NK‐κB signalling pathway to suppress metabolic disorders and apoptosis in NP cells and activates the AKT signalling pathway to stimulate the anabolism of NP cells, which in turn protects and prevents the development of degenerative disc lesions.

Although ghrelin is a short peptide found in the gastrointestinal tract, it is also important for the musculoskeletal system. The age‐dependent nature of ghrelin may be a critical factor in degenerative musculoskeletal disorders. With the acceleration of the ageing process in China and the increasing proportion of the elderly population revealing the potential role of ghrelin as a key target in the treatment of related bone and muscle diseases, there is a need for further research in this area.

## AUTHOR CONTRIBUTIONS

**Jianfeng Sun:** Conceptualization (lead); data curation (equal); formal analysis (equal); investigation (equal); methodology (lead); software (equal); validation (equal); visualization (equal); writing – original draft (lead); writing – review and editing (equal). **Yibo Tan:** Conceptualization (lead); data curation (equal); formal analysis (equal); investigation (equal); methodology (lead); software (equal); validation (equal); visualization (equal); writing – original draft (lead); writing – review and editing (equal). **Jingyue Su:** Formal analysis (equal); investigation (equal); validation (equal); writing – review and editing (equal). **Herasimenka Mikhail:** Formal analysis (equal); investigation (equal); validation (equal); writing – review and editing (equal). **Volotovski Pavel:** Formal analysis (equal); investigation (equal); validation (equal); writing – review and editing (equal). **Zhenhan Deng:** Conceptualization (supporting); funding acquisition (equal); methodology (supporting); project administration (equal); resources (equal); supervision (equal); writing – original draft (supporting); writing – review and editing (equal). **Yusheng Li:** Conceptualization (supporting); funding acquisition (equal); methodology (supporting); project administration (equal); resources (equal); supervision (equal); writing – original draft (supporting); writing – review and editing (equal).

## CONFLICT OF INTEREST STATEMENT

The authors declare that they have no conflict of interest.

## Data Availability

Not applicable.
